# Alpha-Synuclein Cell-to-Cell Transfer and Seeding in Grafted Dopaminergic Neurons *In Vivo*

**DOI:** 10.1371/journal.pone.0039465

**Published:** 2012-06-21

**Authors:** Elodie Angot, Jennifer A. Steiner, Carla M. Lema Tomé, Peter Ekström, Bengt Mattsson, Anders Björklund, Patrik Brundin

**Affiliations:** 1 Neuronal Survival Unit, Department of Experimental Medical Science, Wallenberg Neuroscience Center, Lund University, Lund, Sweden; 2 Center for Neurodegenerative Science, Van Andel Research Institute, Grand Rapids, Michigan, United States of America; 3 ImaGene-iT, Lund, Sweden; 4 Neurobiology Unit, Department of Experimental Medical Science, Wallenberg Neuroscience Center, Lund University, Lund, Sweden; University of Nebraska Medical Center, United States of America

## Abstract

Several people with Parkinson’s disease have been treated with intrastriatal grafts of fetal dopaminergic neurons. Following autopsy, 10–22 years after surgery, some of the grafted neurons contained Lewy bodies similar to those observed in the host brain. Numerous studies have attempted to explain these findings in cell and animal models. In cell culture, α-synuclein has been found to transfer from one cell to another, via mechanisms that include exosomal transport and endocytosis, and in certain cases seed aggregation in the recipient cell. In animal models, transfer of α-synuclein from host brain cells to grafted neurons has been shown, but the reported frequency of the event has been relatively low and little is known about the underlying mechanisms as well as the fate of the transferred α-synuclein. We now demonstrate frequent transfer of α-synuclein from a rat brain engineered to overexpress human α-synuclein to grafted dopaminergic neurons. Further, we show that this model can be used to explore mechanisms underlying cell-to-cell transfer of α-synuclein. Thus, we present evidence both for the involvement of endocytosis in α-synuclein uptake *in viv*o, and for seeding of aggregation of endogenous α-synuclein in the recipient neuron by the transferred α-synuclein. Finally, we show that, at least in a subset of the studied cells, the transmitted α-synuclein is sensitive to proteinase K. Our new model system could be used to test compounds that inhibit cell-to-cell transfer of α-synuclein and therefore might retard progression of Parkinson neuropathology.

## Introduction

People with Parkinson’s disease (PD) exhibit a constellation of motor and non-motor signs and symptoms including bradykinesia, resting tremor, rigidity, depression, and anosmia. The accompanying pathological hallmarks of PD are intracellular proteinaceous deposits termed Lewy bodies and Lewy neurites, which are found both in peripheral organs and in several central nervous system structures [1].

Over the past two decades, intrastriatal neural grafting has been reported to result in long-term relief of some motor symptoms in PD. The disease process, however, continued in the patients’ brains as evidenced by the surprising presence of Lewy bodies and neurites in the grafted neurons [2]–[6]. Currently, it is unclear how these young neurons acquired such pathologies. One provocative explanation for the presence of pathology in the grafted neurons is protein transfer from the host brain to the grafted cells, with subsequent seeding of aggregates in the recipient cells, in analogy to mechanisms operating in prion diseases [7].

The main protein component of Lewy pathology is α-synuclein (αsyn), a synaptic protein with the propensity to misfold and aggregate [8]. The gene encoding αsyn, *SNCA*, is mutated and duplicated or triplicated in rare familial forms of parkinsonism [9]–[14] and single nucleotide polymorphisms in the *SNCA* promoter are linked to sporadic PD [15]. Hence, αsyn is heavily implicated in the pathogenesis of PD. Several studies, both in cultured cells and animal models, have addressed the hypothesis of intercellular transfer of αsyn [16]–[23]. We recently found that human αsyn (huαsyn) transits from cells in the brains of mice expressing huαsyn to naïve neurons grafted into the striatum, in analogy to the mechanism postulated to take place in the grafted PD cases [19]. In cultured cells of human and rodent origin, after its transfer to a recipient cell, αsyn appears to seed aggregates of endogenous αsyn proteins [19], [23]–[27]. Recently, acceleration of huαsyn aggregation in the brain of young, pre-symptomatic transgenic mice, together with earlier onset of neurological symptoms, have been reported after intracerebral inoculation of brain tissue from old transgenic mice affected by the synucleinopathy [22], [28]. Injection of recombinant αsyn fibrils into the brain of young, pre-deposit transgenic mice led to the same effects [22]. These findings are consistent with a “prion-like” propagation of αsyn [22], [28]. Up to this point, however, the whole sequence of events defining the “prion-like” hypothesis, meaning the transfer of αsyn from a donor cell to a recipient neuron, followed by the seeding of the aggregation of the endogenous αsyn from the recipient cell around a core of transferred αsyn, has still not been demonstrated *in vivo*. Additionally, although endocytosis has been suggested as a mechanism involved in the uptake of αsyn from the extracellular space [17], [19], [21], [23], [29], [30], the localization of transferred αsyn in endosomes has not been observed *in vivo*. In this report, we engineered nigral neurons to express huαsyn by injecting a recently developed AAV2/6 viral vector encoding huαsyn (AAV2/6-huαsyn) into the substantia nigra of rats [31]. After several weeks, during which time huαsyn was produced and axonally transported from the nigral cell bodies to the dopamine neuron axon terminals in the striatum [31], we grafted rat embryonic ventral mesencephalic (VM) neurons into the striatum. At several time points after grafting, we sacrificed the rats, processed the brains for immunohistochemistry and screened for the presence of huαsyn in the transplanted neurons. In this model, we detected frequent occurrence of transfer of αsyn from host brain neurons to transplanted tyrosine hydroxylase (TH)-positive neurons. Additionally, we found that transferred huαsyn co-localized with a marker for early endosomes in the grafted neurons. Moreover, we showed that, within the recipient cell, the small immunoreactive dot representing transferred huαsyn was surrounded by a larger area of rodent αsyn-positive signal, suggesting, for the first time, *in vivo* seeding capacity of intercellularly transferred huαsyn. Finally, we report that, at least in the subset of cells we examined, the transmitted huαsyn is sensitive to a proteinase K (PK) treatment, in contrast to the aggregated αsyn proteins that we observed to accumulate in the cell bodies and dystrophic neurites of AAV2/6-huαsyn infected neurons. Taken together, our results could pave the way for future studies to screen for drugs that reduce or block αsyn transfer in whole animals.

## Results

### Neural Grafts Survive in Human α-synuclein-expressing Rat Brain

**Figure 1:**
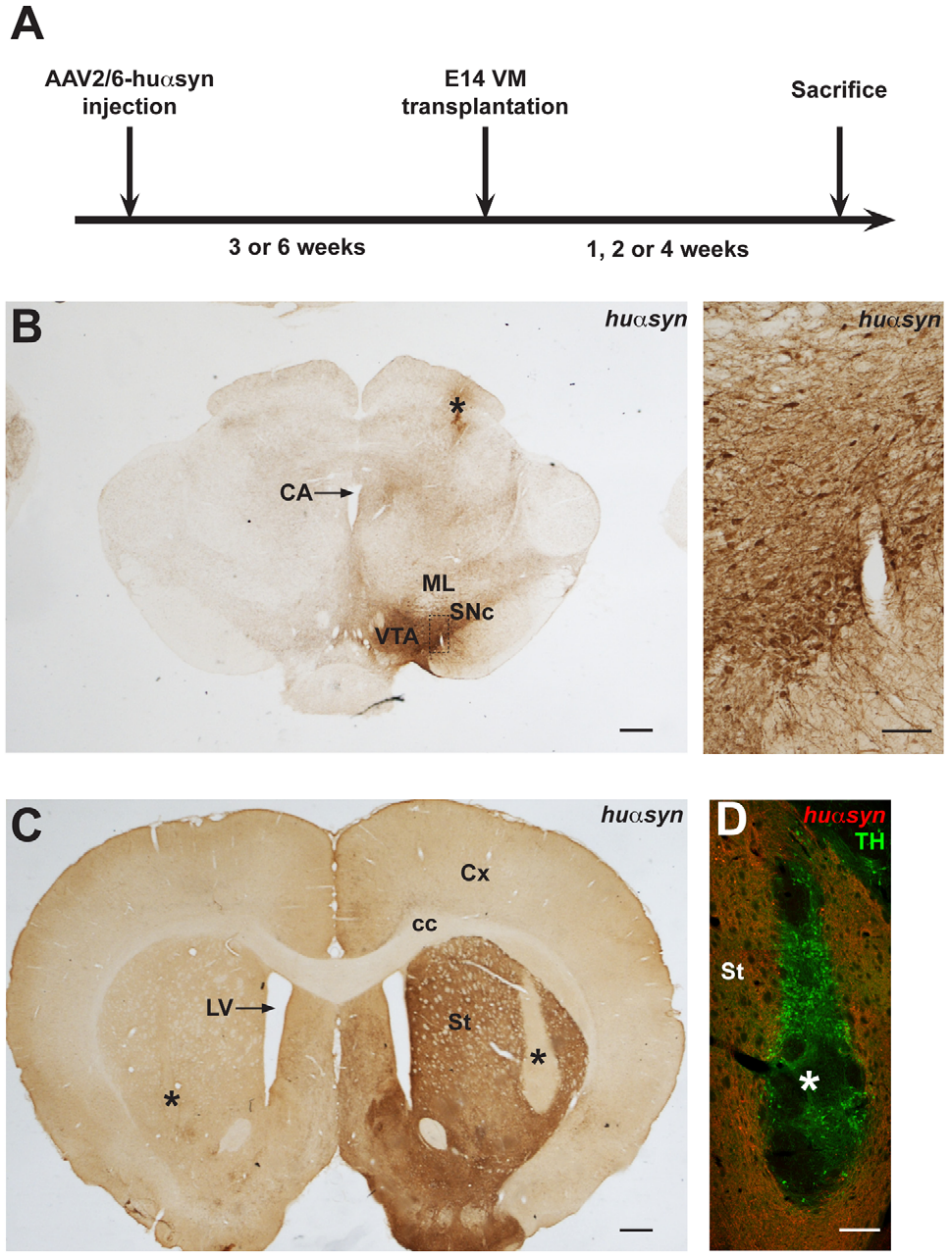
AAV2/6-huαsyn- and transplantation-based rat model for prion-like propagation of α-synuclein. (A) Experimental protocol. Rats were injected in the right substantia nigra with AAV2/6-huαsyn. Three or six weeks later, these rats were transplanted with wild-type rat embryonic day 14 ventral mesencephalic tissue bilaterally in the striatum. Finally, the animals were sacrificed one, two or four weeks after grafting. (B, left) Coronal section at the level of the interpeduncular fossa of a rat ten weeks after AAV2/6 injection. Asterisk marks the injection site. (B, right) High magnification view of the left panel (dashed box) demonstrating expression of huαsyn in the cell bodies of the SNc. (C) Coronal section at the level of the gyrus diagonalis of a rat transplanted with VM tissue six weeks after AAV2/6-huαsyn injection and sacrificed four weeks after grafting. The immunohistochemical analysis with antibodies directed against huαsyn shows the overexpression of this protein in the axon terminals of the right striatum. The center of the bilateral grafts is marked with an asterisk. On the right, the graft is clearly located in the area devoid of signal. (D) Adjacent section from the same animal, subjected to double immunofluorescence with antibodies directed against TH (green) and huαsyn (red). The TH-positive cell bodies of the transplanted neurons are easily distinguished from the surrounding huαsyn-positive host tissue. Here again the asterisk is located in the center of the graft. Abbreviations: CA, cerebral aquaduct; cc, corpus callosum; Cx, cortex; E, embryonic day; LV, lateral ventricle; ML, medial lemniscus; St, striatum; SNc, substantia nigra pars compacta; VM, ventral mesencephalon; VTA, ventral tegmental area. The scale bars for panels B (left), C, and D represent 500 µm, while the scale bar for panel B (right) represents 100 µm.

In order to study αsyn transfer *in vivo* and explore possible transfer mechanisms, we utilized rodent model of huαsyn overexpression, that was recently extensively described in a parallel study [31]. We injected AAV2/6-huαsyn into the right substantia nigra of female Sprague-Dawley rats in order to overexpress huαsyn in the nigrostriatal dopaminergic neurons. Three or six weeks after virus injection, we performed bilateral intrastriatal transplantation of embryonic day 14 VM in the viral-vector transduced rats (Figure 1A). One (n = 6), two (n = 8), or four (n = 12) weeks later, we killed the rats, fixed and sectioned their brains prior to immunostaining.

We first confirmed that the transplanted neurons had survived and were located bilaterally in the center of the striatum of each rat. We observed dense huαsyn immunoreactivity in the cell bodies of the right substantia nigra (Figure 1B) and in the nigrostriatal axon terminals in the striatum (Figure 1C). The areas devoid of huαsyn signal (Figure 1C, asterisks) contained the grafted neurons derived from fetal cells not expressing huαsyn. In another series of sections from each rat, we performed double immunofluorescence for TH and huαsyn, in order to visualize the individual TH-positive neurons within the huαsyn-positive host tissue (Figure 1D). As dopaminergic neuron cell bodies are normally not found in the striatum, all TH-expressing somata we identified in the striatum were grafted neurons. The number of surviving TH-expressing neurons has previously been reported to be unchanged in intrastriatal grafts, one to four weeks after the surgery procedure [32]–[34]. Thus, when we sampled our animals for stereology-based counting of the total number of TH-positive cells within the graft, we randomly selected six out of the 12 animals transplanted three weeks after viral injection and six out of the 14 animals grafted six weeks after AAV2/6 transduction, without taking in account the survival time after transplantation. We found a total number of surviving grafted dopaminergic cells of 2438±296 and we did not detect any effect of the severity of the synucleinopathy at the time of grafting on the survival of transplanted dopamine neurons (Figure S1, 2580±440 and 2296±428 for the rats transplanted three and six weeks after viral injections, respectively). Huαsyn-positive axon terminals derived from the host nigrostriatal neurons surrounded the grafts (Figure 1D). A few of huαsyn-positive axons traversed the host/graft border and reached the periphery of the implants.

### α-Synuclein Transfers from Host Brain to Grafted Neurons

**Figure 2:**
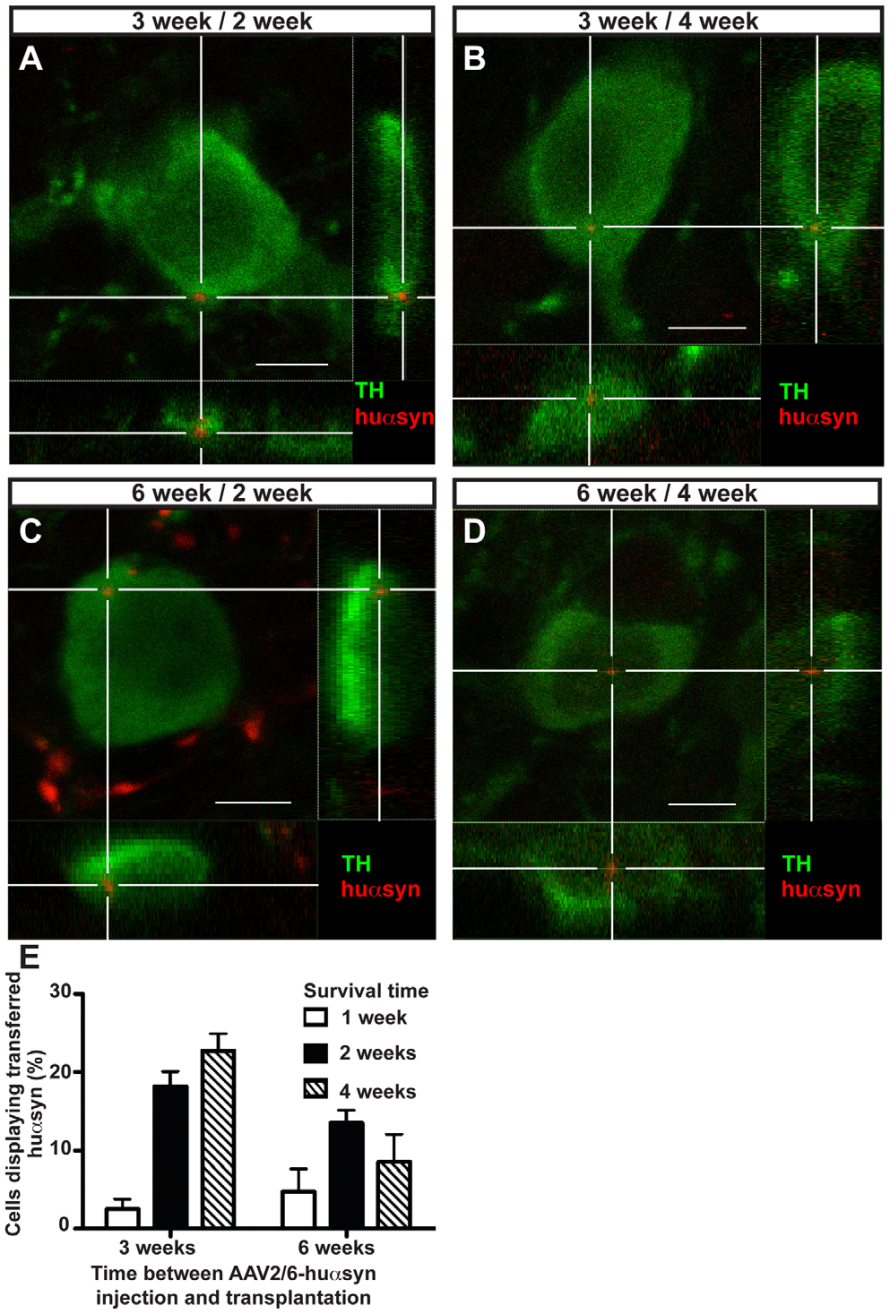
The propagation of human α-synuclein from host tissue to transplanted dopaminergic neurons is a disease stage- and time-dependent process. (A-D) Confocal three-dimensional reconstructions of grafted TH-positive neurons (green) displaying intracellular puncta of transferred huαsyn (red), which are marked by the white cross. Reconstructed orthogonal projections are presented as viewed in the x-z (bottom) and y-z (right) planes. These cells were detected in rats transplanted either three (A, B) or six (C, D) weeks after AAV2/6-huαsyn injection and sacrificed two (A, C) or four (B, D) weeks after grafting. (E) Quantification of the percentage of transplanted TH-expressing cells that show intracellular puncta of huαsyn in each experimental group. Each bar represents the number of transferred huαsyn-positive cells compared to the total TH-positive cells counted in each group. We randomly sampled more than 20 TH-positive cells per rat, in three to seven rats per group. Scale bars equal 6 µm.

We studied 26 rat brains that displayed the expected huαsyn-immunoreactivity and had appropriately located TH-positive grafts in the center of the striatum. Using an epifluorescence microscope, we observed several hundred TH-immunoreactive grafted neurons in which small puncta of huαsyn immunoreactivity appeared to be located. As expected, we never detected huαsyn signal in any TH-positive neurons in the transplant injected into the left striatum, which is consistent with the fact that we had transduced host neurons with AAV2/6-huαsyn in the nigrostriatal pathway only on the right side. We performed confocal microscopy on at least 20 (Figure S2) randomly selected TH-immunoreactive neurons per rat and collected three-dimensional reconstructions of each of these cells. In some cases, huαsyn-positive puncta were located inside the TH-positive cells. For other grafted TH-expressing cells, we found the huαsyn to be located exclusively immediately adjacent, as would be the case if the observed huαsyn immunoreactivity were inside a terminal of the host nigrostriatal pathway. Finally, in several cases we also found such huαsyn-immunoreactive profiles immediately outside neurons displaying clear intracellular huαsyn immunoreactivity. Thus, the grafted neurons we scored as positive for huαsyn transfer clearly displayed intracellular huαsyn signal. Figure 2A-D shows images obtained from rats of the 3 week/2 week, 3 week/4 week, 6 week/2 week and 6 week/4 week groups, illustrating representative TH-positive neurons (green) containing intracellular huαsyn puncta (red). Next, we quantified the frequency of grafted TH-positive neurons exhibiting huαsyn transfer in each group by calculating the percentage of TH-positive neurons that displayed one or more intracellular huαsyn puncta (Figure 2E, S2). We found that the proportion of grafted cells displaying huαsyn uptake depends significantly on the time after transplantation (2 way ANOVA, main effect of time after grafting, F = 8.93, p<0.05) and on the duration of time between virus injection and grafting (2 way ANOVA, main effect of the time between AAV2/6-huαsyn injection and transplantation, F = 4.85, p<0.05). Importantly, the time between virus injection and grafting and the time after transplantation, or survival time, interact to influence the percentage of cells exhibiting huαsyn signal (2 way ANOVA, interaction effect, F = 3.81, p<0.05). Furthermore, a duration of three weeks between viral injection and transplantation combined with four weeks of survival time results in a high percentage of cells displaying transferred huαsyn (22.7±2.23%). These results indicate that the time elapsed after grafting and the stage of the synucleinopathy, which becomes more severe with time after virus injection [31], both influence the likelihood that we observe transferred huαsyn.

### Transferred α-synuclein Colocalizes with Endosomal Marker

**Figure 3:**
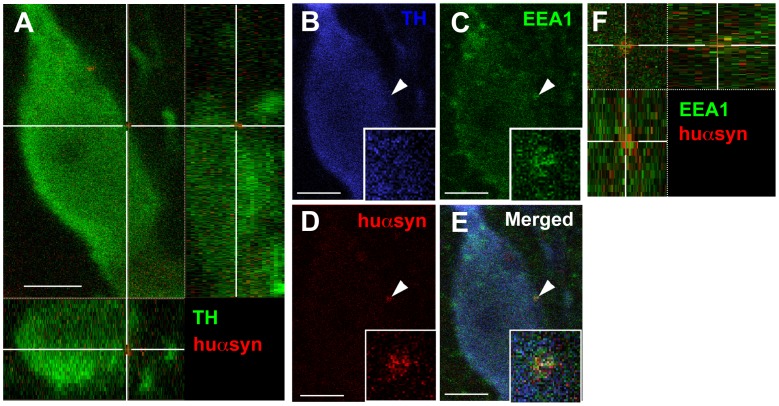
Endocytosis is involved in human α-synuclein intercellular transfer. (A) Confocal three-dimensional reconstruction showing the intracellular location of a transferred huαsyn punctum (red) within a TH-positive transplanted neuron (green) from the 3 week/4 week group. (B-D) Triple staining with antibodies raised against TH (B, blue), EEA1 (C, green) and huαsyn (D, red) shows the colocalization of this transmitted huαsyn punctum with EEA1 (arrowheads). Panel E is a merged picture of (B), (C), and (D). The insets in (B), (C), (D) and (E) are higher magnifications of the huαsyn- and EEA1-positive punctum. (F) High magnification three-dimensional reconstruction of the huαsyn- (red) and EEA1-positive (green) punctum. Scale bars represent 6 µm.

Previous *in vitro* studies have suggested that endocytosis is involved in the uptake of αsyn from the extracellular space [19], [23]–[27]. The transferred huαsyn dots were very heterogeneous in terms of size and intracellular localization. Only a few TH-positive grafted neurons showed a huαsyn intracellular signal compatible with endocytic localization. This is why we developed a rigorous stripping protocol, leading to complete removal of antibodies bound to the sections during an earlier round of staining. This method allowed us to return to the specific transferred huαsyn dots identified in our first round of screening and examine if they co-localized with endosomal markers, rather than performing a “blind” triple staining with antibodies directed against TH, huαyn and endosomal marker on a new section. Before applying this technique routinely, we confirmed that no fluorescence signal from the first round of staining remained on the stripped sections. Moreover, we determined that the stripping procedure did not damage the huαsyn antigens, so that we were still able to detect the transferred huαsyn dots in the second round of staining (Figure S3). We then triple-stained the sections with TH, huαsyn, and EEA1 antisera using different fluorochromes to detect each antibody. In Figure 3A we present a TH-positive cell containing a huαsyn-immunoreactive punctum with an intracellular, juxtamembrane localization, which led us to suspect that it might have been recently taken up. Indeed, upon stripping and restaining, we found that the early endosome marker EEA1 co-localized with the transferred huαsyn-immunopositive punctum in the TH-positive cell (Figure 3B–F), suggesting endocytic localization for huαsyn taken up *in vivo*.

### Transferred α-synuclein Seeds Aggregation *in vivo*

**Figure 4:**
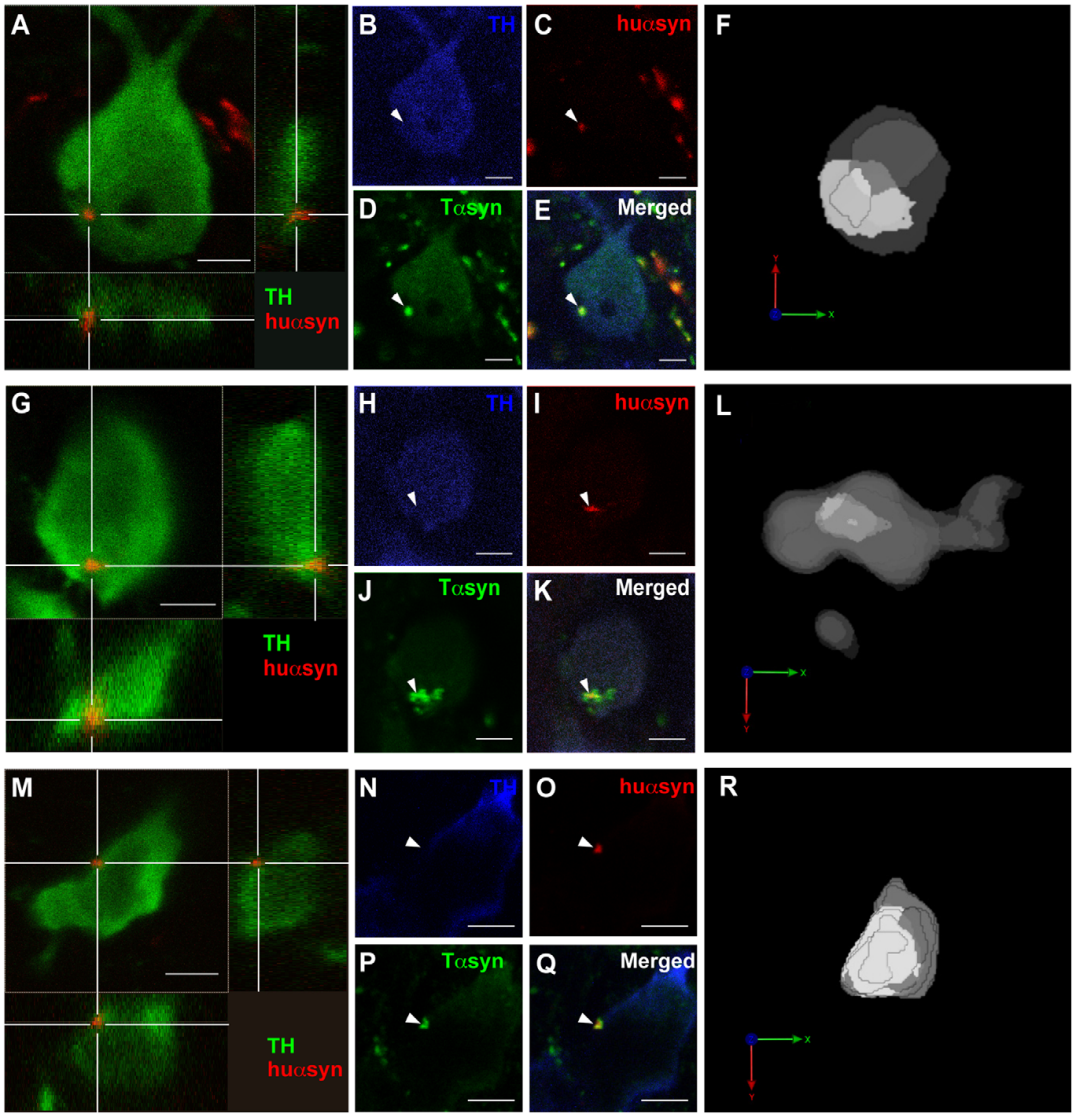
Transferred human α-synuclein seeds the aggregation of rat α-synuclein in the recipient cell. (A, G, M) Confocal three-dimensional reconstructions of three transplanted TH-expressing (green) cells positive for huαsyn (red) transfer. Panel A shows a TH-positive cell from the 6 week/4 week group, panel G displays a TH-expressing cell from the 6 week/2 week group and panel M corresponds to a TH-expressing cell from the 3 week/4 week group. (B-E, H-K, N-Q) Triple staining with antibodies directed against TH (B, H, N, blue), huαsyn (C, I, O, red) and both huαsyn and rodent αsyn (Tαsyn, D, J, P, green) shows the larger area stained with the total αsyn antibody compared to the huαsyn signal alone (arrowheads). The E, K and Q panels are merged pictures of (B), (C), and (D); (H), (I) and (J); and (N), (O) and (P), respectively. (F, L, R) Three-dimensional reconstructions with opaque rendering showing the core of huαsyn in light grey whereas the total αsyn is in dark grey. This view underlines the embedding of huαsyn nucleus within a shell of total αsyn signal. The orientation is indicated by the coordinate system with the x axis in green, y axis in red and z axis in blue. Scale bars represent 5 µm.

We found several TH-positive cells containing intracellular huαsyn dot located further away from the outer membrane and of bigger size, clearly different from the smaller huαsyn puncta with juxtamembrane localization we described above. Interestingly, we observed that the cytoplasm immediately surrounding these puncta often exhibited low levels of TH staining (Figure 4A and G). For these sections, we stripped the two bound antibodies and reprobed with three antibodies directed against TH, huαsyn, and total αsyn, as the large size of these huαsyn accumulations led us to suspect that they could be able of seeding activity. Indeed, analogous to previous findings in cell culture models of αsyn uptake [19], [23]–[27], we found a core of huαsyn surrounded by a larger area of total αsyn-immunoreactivity (Figure 4B–E, H-K, N-Q). Analysis with image processing software provided a three-dimensional reconstruction, which further supported the embedding of a nucleus of transferred huαsyn within a shell of total α-syn-positive signal (Figure 4F, L, R). This result argues for a specific interaction between huαsyn and rat αsyn and suggests a seeding activity of transferred huαsyn on rat αsyn proteins within the recipient grafted neuron.

### Transferred α-synuclein is PK-sensitive and Non-phosphorylated

In order to characterize biochemically the huαsyn protein that had transferred from the brain of AAV2/6-huαsyn injected rats to the grafted dopaminergic neurons, we assessed its aggregation and phosphorylation state.

**Figure 5:**
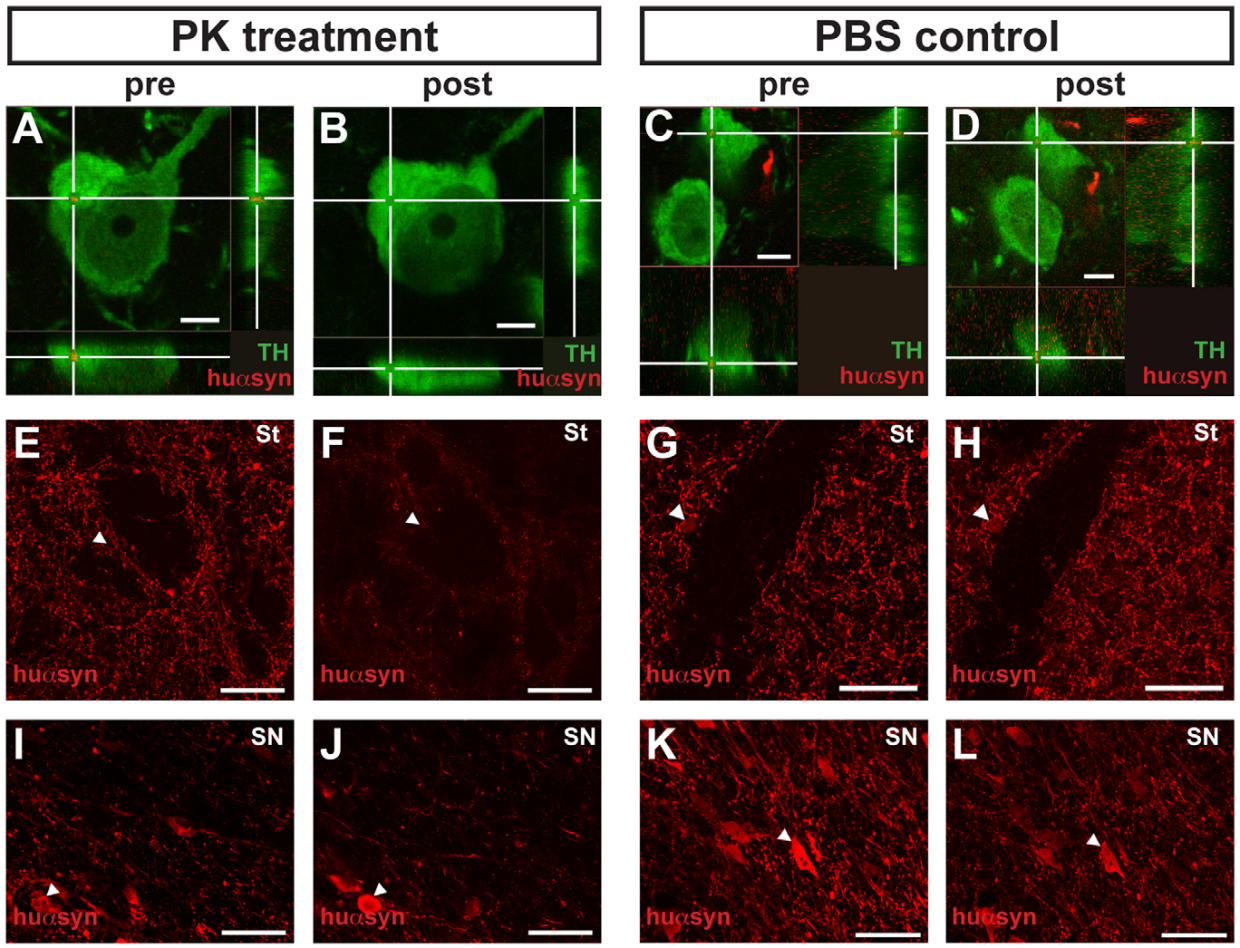
Transferred human α-synuclein is proteinase-K sensitive. (A-D) Confocal three-dimensional reconstructions of two TH-positive neurons (green) transplanted in rats from the 3 week/4 week group. (A) and (C) show the intracellular localization of a transferred huαsyn punctum (red) within these neurons. (B) and (D) depict the same neurons as (A) and (C); however, the antibodies were stripped from (A) and (C), and the sections were then treated with PK (B) or PBS (control, D), then re-stained. Whereas the huαsyn dot is still present in the cell treated with PBS (compare D to C), the transferred huαsyn has been dissolved by the PK treatment (compare B to A). The white cross marks the transferred huαsyn punctum (A, C, D) or its localization prior to the PK treatment (B). (E-H) and (I-L) show confocal planes of coronal sections from, respectively, the striatum and the subtantia nigra of rats of the 3 week/4 week group, stained with antibodies directed against huαsyn. (F), (H), (J) and (L) correspond to the same areas as (E), (G), (I) and (K), respectively; however, the antibodies were stripped from (E), (G), (I) and (K), and the sections were then treated with PK (F, J) or PBS (control, H, L) and re-stained. The arrowheads indicate landmarks that have been used to match the areas of interest before and after treatment. Note the apparent attenuation of signal in total area stained after PK treatment (compare F to E and J to I) and the very high similarity between the staining before and after PBS control treatment (compare H to G and L to K). The sections in (A-B), (E-F), (I-J) on one side and (C-D), (G-H) and (K-L) on the other side have been processed simultaneously, in the exact same conditions. Abbreviations: PK, proteinase K; St, striatum; SN, subtantia nigra. Scale bars represent 5 µm (A-D) or 50 µm (E-L).

First, we optimized a PK treatment protocol. After testing several conditions, we found that exposing the sections to 10 µg/mL PK for 10 minutes at room temperature resulted in digestion of most of the huαsyn in the striatal axonal terminals of the nigral neurons infected with AAV2/6-huαsyn (Figure 5E, F). By contrast, the aggregated forms of huαsyn found both in abnormal swellings (or varicosities) of dystrophic neurites in the striatum (Figure 5F) and in cell bodies in the substantia nigra (Figure 5I, J) remained and were stained with the antibody directed against huαsyn. Then, we applied the exact same PK conditions to stripped sections that we previously had found to show grafted TH-positive neurons containing intracellular puncta immunoreactive for huαsyn. After re-staining with antibodies against TH and huαsyn, we found that the huαsyn immunoreactive dots were no longer visible inside the 6 grafted neurons we examined (Figure 5A, B). As a control we treated other sections with PBS instead of PK. As predicted, we found that the PBS treatment did not affect the immunoreactivity of the transmitted huαsyn (Figure 5C, D). Taken together we showed that the transmitted huαsyn we observed in 6 cells was sensitive to PK treatment, indicating that non-aggregated forms of huαsyn can transfer between cells *in vivo*.

We also assessed the phosphorylation state of the transferred huαsyn. As expected, we found that antibodies specifically directed against α-syn phosphorylated on serine 129 recognize the huαsyn that accumulates in TH-positive varicosities in the striatum (Figure S4A-D) and cell bodies in the substantia nigra (Figure S4E-H) of AAV2/6-huαsyn transduced neurons. However, we did not detect any colocalization between the transmitted huαsyn with α-syn phosphorylated on serine 129 within TH-expressing transplanted neurons.

In brief, our results suggest that at least some of the huαsyn forms that transfer to transplanted dopaminergic neurons are not aggregated or phosphorylated. In contrast, we found that a significant portion of the huαsyn that accumulates within dystrophic neurites and nigral neuronal cell bodies following infection by AAV2/6-huαsyn, is contained in PK-resistant aggregates that include αsyn phosphorylated on serine 129.

## Discussion

In this report, we show intercellular transfer of huαsyn from host brain cells to naïve neurons grafted into rat striatum three or six weeks after AAV2/6-huαsyn virus transduction into the substantia nigra, in accordance with a recent report from Kordower et al describing a similar AAV2/6-huαsyn model. In the study from Kordower and coworkers, rats subjected to AAV2/6-GFP transduction were used as negative controls and transfer of GFP from the host brain to grafted neurons was never observed [35]. In our study we extend these findings by varying the time between virus injection, neural grafting, and sacrifice, thus modeling the different stages of human synucleinopathy from mild to severe. Three weeks after AAV2/6-huαsyn virus injection into the rat striatum, the animals have been described to be in a “presymptomatic” stage and display normal performance in the motor test and no or limited nigral neuron loss [31]. Six weeks post-viral transduction corresponds to an early symptomatic stage with degeneration of 50% of the nigral neurons [31]. Finally, at eight weeks post-viral transduction (a time point only reached by the rats grafted six weeks after transduction), the motor deficits are fully developed and up to 70% of the nigral dopaminergic neurons are lost [31]. Of relevance to the aforementioned neural transplantation studies and subsequent autopsies conducted in humans, we examined the effect of both the disease severity at the time of grafting and the survival time after transplantation on the likelihood of observing grafted cells that had taken up huαsyn (Figure 2). It has previously been suggested that the presence of Lewy bodies in grafted neurons is a time-dependent process, with the percentage of neurons displaying Lewy bodies being higher in older grafts than in younger transplants [2], [6]. Furthermore, a minimum duration between grafting and death around one decade appears to be necessary in order to support the presence of Lewy bodies in grafted neurons [2]. Our study supports these claims as we show a time-dependent increase in the percentage of TH-positive neurons exhibiting intracellular huαsyn puncta (Figure 2). At the final time point in the group grafted at a later stage of synucleinopathy, the percentage of neurons exhibiting huαsyn puncta decreases. This result could be due to death of some of the grafted neurons that have taken up huαsyn. Alternatively, neurons that have taken up huαsyn are able to degrade the imported protein and the recruitment of new neurons taking up huαsyn simply decreases with time due to the degeneration of striatal dopamine terminals and thus the decrease of the huαsyn input. Indeed, we recently reported, in rats injected with the AAV2/6-huαsyn virus under the exact same conditions, a reduction of the TH-expressing striatal fiber density to 69% (compared to staining in contralateral non-injected side) after three weeks, which was maintained after five weeks and further decreased to 42% after eight weeks [31].

The involvement of endocytosis in the uptake of αsyn into many cell types has been demonstrated in cell culture [17], [19], [21], [23], [29], [30]. Most of these studies have utilized strategies to inhibit endocytosis to reach their conclusions. In neurons grafted *in vivo,* we now show a particular endocytic compartment where transmitted αsyn can be found (Figure 3). Interestingly, we did not observe this endocytic localization in every TH-expressing cell that displayed transferred huαsyn, which suggests that the other localizations (as seen in Figure 2) show huαsyn that has already escaped from a vesicle.

αSyn aggregates are seen in several neuron and glial types in synucleinopathies [36]–[40]. Moreover, the capacity of glial cells to take up αsyn has been recently demonstrated *in vitro* [41] and *in vivo* [21]. Thus, it will be important to explore mechanisms of αsyn uptake not only into dopaminergic neurons, but also into other types of cells. Interestingly, Kordower and colleagues recently demonstrated uptake of huαsyn into both grafted TH-positive and non-TH positive cells in the study mentioned above [35].

In our study, we show a core of intracellular huαsyn surrounded by endogenous rat αsyn (Figure 4), suggesting for the first time that αsyn, which has transferred between cells, can act as a seed attracting endogenous αsyn produced by the rat neuron. These findings are consistent with results from different *in vitro* cell models [19], [23]–[27] and for the first time extend this observation into an *in vivo* setting. However, it remains unclear how αsyn gains access from the endocytic compartment or directly from the extracellular space to the cytoplasm of cells in order to permissively template endogenous αsyn. Further studies are needed to clarify this important step in the process.

Finally, we report that at least some of the αsyn that has transferred from host brain cells to transplanted neurons is sensitive to a PK digestion protocol that does not destroy αsyn aggregates formed in neurons transduced by the viral vector. This suggests that αsyn can transfer between cells in a non-aggregated form and that once in the recipient cell, it does not necessarily form aggregates. In the rare cases where we observed that transferred huαsyn attracted endogenous rat αsyn (e.g. Figure 4), it is conceivable that the resulting αsyn assemblies would be more resistant to PK. However, in this study we did not have the opportunity to systematically examine if such cells lose their αsyn immunoreactivity following PK digestion. In the future it will be crucial to identify which αsyn specie(s) transfer(s) the most efficiently between neurons and under what conditions the transferred αsyn will act as a seed for aggregation in the new host neuron. Understanding these events will be essential in developing disease-modifying therapeutics which interfere with the spreading of synucleinopathy during PD progression.

## Materials and Methods

### Animals

Sprague-Dawley female rats (225 g) were purchased from Charles River Laboratories. The rats were housed two or three per cage under a 12-hour light/12-hour dark cycle with ad libitum access to food and water. The housing of the animals and all procedures were carried out in accordance with international guidelines and were approved by the Malmö-Lund Ethical Committee for Animal Research (Permit Number: M162-10).

### Vector Preparation and Injection

We utilized an AAV2/6 vector in which the expression of the human wild-type αsyn transgene was driven by the synapsin 1 promoter and enhanced using a woodchuck hepatitis virus post-transcriptional regulatory element. Vector production was performed as previously described [31], [42]. Briefly, a transfer plasmid carrying AAV2 Inverted Terminal Repeats encoding human wild-type αsyn downstream to the synapsin 1 promoter was generated and transfected into human embryonic kidney 293 cells using the calcium-phosphate method, and included the packaging plasmid pDP6 encoding the AAV6 capsid proteins [43], [44]. The cells were lysed with buffer (50 mM Tris, 150 mM NaCl, pH 8.4) and by performing freeze-thaw cycles in dry ice/ethanol bath. The crude lysates were purified first by ultracentrifugation (1.5 hours at 350 000×g at 18°C) in a discontinuous iodixanol gradient, and the virus-containing fractions were purified with ion-exchange chromatography using FPLC. Genome copy titer was determined using real-time quantitative PCR. The genome copy titer used in the injections was 7.0×10^12^ genome copies/mL.

We performed all surgical procedures under general anesthesia using a 20∶1 mixture of fentanylcitrate (Fentanyl) and medetomidin hypochloride (Dormitor) (Apoteksbolaget, Sweden) injected intraperitoneally. Rats were placed in a stereotaxic frame (Stoelting) and vector solutions were injected using a 10 µL Hamilton syringe fitted with a glass capillary (outer diameter of 100–200 µm). 3 µL of the AAV2/6-huαsyn vector solution were infused at a rate of 0.2 µL/min and the needle was left in place for an additional 3 min period before it was slowly retracted at a rate of 1 mm per minute. We injected AAV2/6-huαsyn vector unilaterally on the right side, above the substantia nigra, at the following coordinates (flat skull position, coordinates relative to bregma and dural surface): antero-posterior: −5.3 mm, medio-lateral: −1.7 mm, dorso-ventral: −7.2 mm.

### Grafting Procedure

We dissected the ventral mesencephalon (VM) from embryonic day 14 rats in cold HBSS-Ca^2+/^Mg^2+^ (Invitrogen) as previously described [45]. We incubated the VM pieces in HBSS-Ca^2+^/Mg^2+^ containing 0.1% trypsin and 0.05% DNase for 15 minutes at 37°C. After rinsing, the VM tissues were mechanically dissociated into a cell suspension containing a mixture of single cells and small aggregates. The number of viable cells was estimated based on Trypan blue (Sigma-Aldrich) exclusion and found to be over 95%. After centrifugation (180×g, 10 minutes, 4°C), the supernatant was removed and the volume was adjusted to give a suspension equivalent to two VMs/animal in HBSS-Ca^2+^/Mg^2+^. The cells were stored on ice during the transplantation procedure. Either three or six weeks after injection of the AAV2/6-huαsyn vector, each rat received bilateral intrastriatal transplants (3 µL, equivalent to about one VM in each striatum) using a Hamilton syringe (coordinates, AP: 0.5 mm; ML: +/−3.5 mm; DV: −5.0, −4.5 mm relative to bregma and dural surface).

### Immunohistochemistry and Microscopy

One, two, or four weeks after grafting, we anesthetized the rats with sodium pentobarbitone and perfused them transcardially with 0.9% saline followed by 4% paraformaldehyde (PFA) in phosphate buffer. We removed the brains and post-fixed them in PFA for 24 hours before placing them in 20% sucrose until sectioning. We cut 40 µm thick free-floating sections on a freezing microtome and immunostained them with primary antibodies against TH (raised in rabbit, 1∶1000; PelFreeze or raised in sheep, 1∶1000; Abcam) and/or specific antisera to huαsyn (raised in mouse, 1∶2000; Abcam), to total αsyn (raised in rabbit, 1∶500; Chemicon), to αsyn phosphorylated on serine 129 (raised in rabbit, 1∶2000; Abcam), to early endosome antigen 1 (EEA1; raised in rabbit, 1∶500; Abcam). For detection of antibodies directed against TH or huαsyn with the chromogen 3,3′-diaminobenzidine (DAB), we incubated the sections in, respectively, biotinylated goat anti-rabbit or horse anti-mouse serum (1∶200; Vector Laboratories) and then processed them for a standard peroxidase-based method (Vectastain ABC kit and DAB kit; Vector Laboratories). For immunofluorescence staining, either Cy2-, Cy3-, Cy5-conjugated antisera (Jackson Immunoresearch Laboratories) or AlexaFluor 488-, AlexaFluor 555-, AlexaFluor647-conjugated antisera (Molecular Probes) were used. After staining, we mounted the sections onto gelatin-coated slides with polyvinyl alcohol medium (Sigma). We analyzed the sections either with a conventional epifluorescence microscope (Eclipse 80i microscope; Nikon) or with a confocal microscope (Leica TCS SL model,equipped with GreNe and HeNe lasers or Zeiss LSM 510, equipped with Ar and HeNe lasers).

### Stereological Counting

We quantified the survival of transplanted TH-positive neurons using Visiopharm Integrator System software (Visiopharm A/S, Horsholm, Denmark) and an Olympus BX50 microscope. We included every eight section of striatum containing grafted cells. Identical quantification parameters were used in all sampling: objective 40x, fraction = 100%, counting frame size x = 200 µm and y = 150 µm. Parameters were chosen to minimize the coefficient of error to <0.10. The total number of grafted cells were calculated using the fractionator formula N = Q * 1/hsf * 1/asf * 1/ssf, where N is the total number of TH-positive cells, Q is the sum of the cells counted, hsf is the height sampling fraction, asf is the area sampling fraction, and ssf is the slice sampling fraction.

### Antibody Stripping and Restaining

In order to reprobe previously immunostained sections with different antibodies, we removed the coverslips from the mounted sections by shaking the slides in phosphate-buffered saline overnight at room temperature. Next, we confirmed the absence of mounting media from the sections. Then, we applied a mild antibody stripping solution (Re-blot Plus; Millipore) to the mounted sections for 15 minutes, at 4°C, with gentle shaking, in order to clear the antibodies bound to the sections during the first staining. At the end of this period we stopped the reaction with several washes in phosphate-buffered saline (PBS). We confirmed the complete removal of signal by examining the sections on both the epifluorescence and confocal microscopes. Finally, the sections were gently removed from the slides mechanically, after they were loosened by PBS washes, and the staining process on free-floating sections was repeated as indicated above.

### Proteinase K Treatment

After a stripping step described above, sections were removed from the slides and incubated in proteinase K (PK, Invitrogen) at 10 µg/mL in PBS or in PBS alone as a control, at room temperature for 10 minutes. The sections were then washed in PBS and re-stained according to the protocol already described in the “Immunohistochemistry and microscopy” section.

### 3-D Rendering of the Interaction between Transferred Huαsyn and Endogenous Rat αsyn

In order to visualize more clearly that in some instances huαsyn was surrounded by endogenous total αsyn in grafted neurons, we processed selected confocal stacks for 3D-rendering of the immunofluorescent structures. Prior to 3D-rendering, boundaries of immunofluorescent structures were emphasized by thresholding. Briefly, we cropped stacks to 64×64 or 128×128 pixels containing the regions of interest (ROI). We extracted red (huαsyn) and green (total αsyn) channels, transformed them to 8-bit greyscale, median filtered (radius 3 pixels), resampled to 256×256 pixels (bicubic smoothing), and segmented into binary images by thresholding (Adobe CS5, Photoshop). We then used the resulting stacks of binary images for 3-D-rendering, using 3-D opacity algorithms (Volocity 6.0, Improvision).

### Statistics

Throughout our paper, the groups of animals are labeled with “x week/y week” where x is the number of weeks between AAV2/6 injection and grafting and y is the number of weeks between grafting and perfusion. The group 3 week/1 week contains three animals, 3 week/2 week contains four animals, 3 week/4 week contains five animals, 6 week/1 week contains three animals, 6 week/2 week contains four animals and 6 week/4 week contains seven animals. We report means plus or minus standard error of the mean. A *p* value of ≤0.05 was taken as significant for all statistical tests, as detailed in the figure legends.

## Supporting Information

Figure S1 **The severity of the synucleinopathy at the time of grafting does not affect the survival of dopaminergic cells within the graft.** Stereology analysis revealed no difference in the total number of TH-expressing cells in the striatal graft between animals transplanted at three (n = 6, 2580±440) or six (n = 6, 2296±428) weeks post-viral transduction. The error bars represent SEM.
(TIF)

Figure S2 **Raw data for the quantification of human** α-**synuclein transfer in grafted AAV2/6-hu**α**syn injected rats.** This table contains all of the animals analyzed in each time-related group (delay between AAV2/6-huαsyn injection and grafting, delay between grafting and sacrifice). The columns show the total number of cells imaged per animal, and among these cells, the number of cells scored positive for huαsyn transfer, i.e. the number of TH-expressing cells containing a huαsyn-positive punctum.
(TIF)

Figure S3 **Controls for the stripping procedure.** (A-C) Confocal planes of a transplanted TH-expressing (green) cell positive for transferred huαsyn (red) punctum (arrowhead). This cell belongs to a section from an animal from the 3 week/2 week group, first processed with rabbit antibody directed against TH and mouse antibody directed against huαsyn, detected with the secondary antibodies Cy2-labeled donkey anti-rabbit and Cy3-labeled donkey anti-mouse, respectively. (A), (B) and (C) show the fluorescence signal captured, respectively, on the green, red and blue channels of the confocal microscope. (D-F) After a stripping procedure performed according to the protocol described in Material and Methods, the same section was re-stained with sheep anti-TH antibody and the same mouse anti-huαsyn antibody as before, then detected with the secondary antibodies Cy5-labeled donkey anti-sheep and Cy3-labeled donkey anti-mouse, respectively. The same cell as the one depicted in (A-C) was imaged in the three channels of the confocal microscope (D-F). (D) is the signal detected in the green channel and shows the absence of remaining fluorescence from the previous staining. (E) is the signal in the red channel and shows that after this stripping/re-staining procedure, the transferred huαsyn punctum (arrowhead) can still be detected. (F) is the signal in the blue channel and demonstrates that after stripping, the TH can be detected with an antibody different from the one used in the first staining. Scale bars, 5 µm.
(TIF)

Figure S4 **AAV2/6-huαsyn injected rats express phosphorylated α-synuclein both in the subtantia nigra and in the striatum.** (A-H) Coronal sections from the striatum (A-D) or the substantia nigra (E-H) of a rat from the 3 week/4 week group, stained with antibodies directed against α-syn phosphorylated on serine 129 (green, pαsyn, A, E), human α-syn (red, B, F) and TH (blue, C, G). (D) and (H) are the merged pictures of (A), (B), (C) and (E), (F), (G), respectively. The arrowheads in (A-D) mark varicosities co-expressing pαsyn, huαsyn and TH. The insets in (A-D) and (E-H) show high magnification pictures of, respectively, a striatal varicosity and nigral dopaminergic neurons co-expressing the three markers. Abbreviations: St, striatum; SN, subtantia nigra. The scale bars equal 200 µm.
(TIF)
