# Supplementary figures and images for: Alpha-Synuclein Cell-to-Cell Transfer and Seeding in Grafted Dopaminergic Neurons In Vivo
Source: PLoS One. 2012 Jun 21;7(6):e39465. doi: 10.1371/journal.pone.0039465 (PMC3380846; doi:10.1371/journal.pone.0039465)

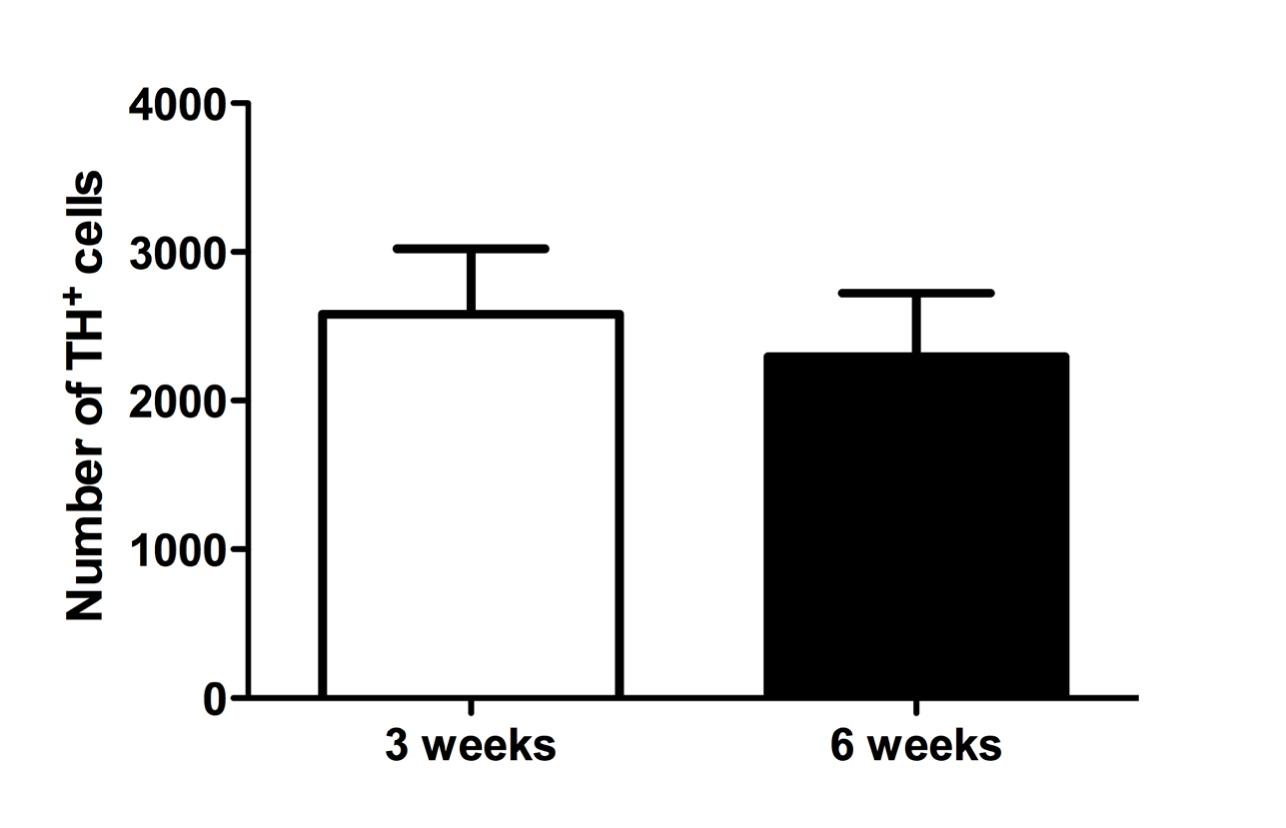

Supplement: Figure S1 — The severity of the synucleinopathy at the time of grafting does not affect the survival of dopaminergic cells within the graft. Stereology analysis revealed no difference in the total number of TH-expressing cells in the striatal graft between animals transplanted at three (n = 6, 2580±440) or six (n = 6, 2296±428) weeks post-viral transduction. The error bars represent SEM. (TIF) [file pone.0039465.s001.tif]

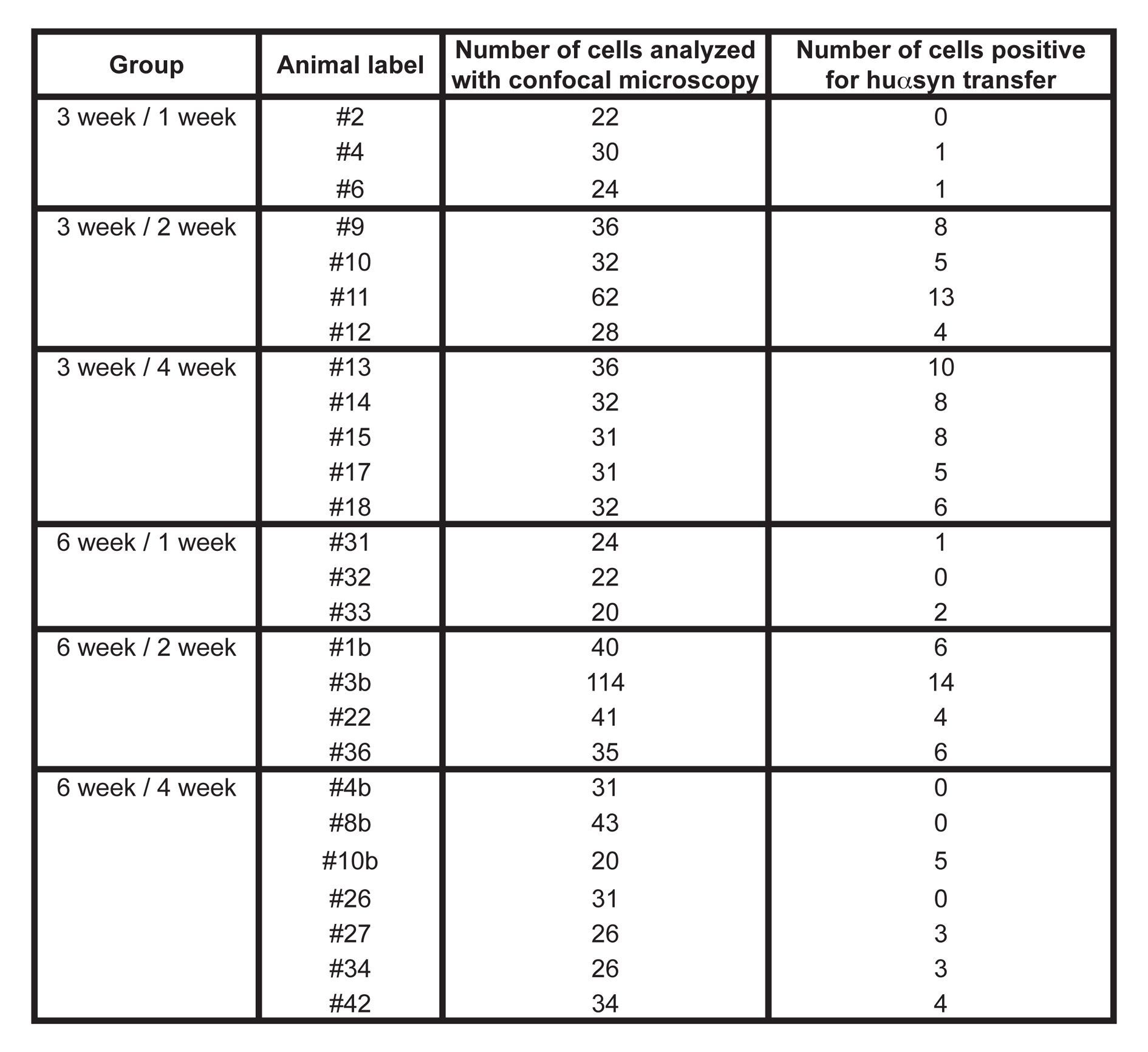

Supplement: Figure S2 — Raw data for the quantification of human α-synuclein transfer in grafted AAV2/6-huαsyn injected rats. This table contains all of the animals analyzed in each time-related group (delay between AAV2/6-huαsyn injection and grafting, delay between grafting and sacrifice). The columns show the total number of cells imaged per animal, and among these cells, the number of cells scored positive for huαsyn transfer, i.e. the number of TH-expressing cells containing a huαsyn-positive punctum. (TIF) [file pone.0039465.s002.tif]

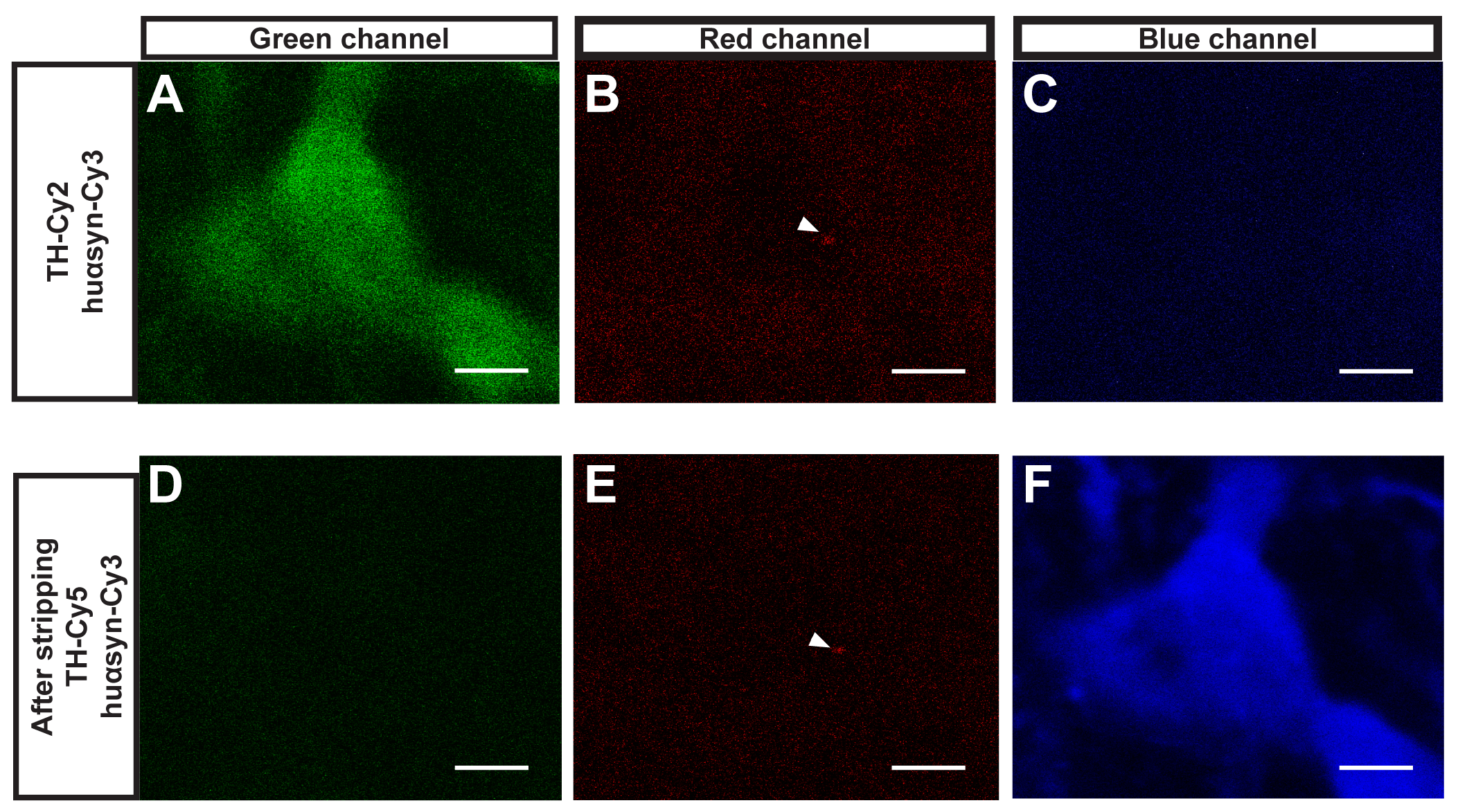

Supplement: Figure S3 — Controls for the stripping procedure. (A-C) Confocal planes of a transplanted TH-expressing (green) cell positive for transferred huαsyn (red) punctum (arrowhead). This cell belongs to a section from an animal from the 3 week/2 week group, first processed with rabbit antibody directed against TH and mouse antibody directed against huαsyn, detected with the secondary antibodies Cy2-labeled donkey anti-rabbit and Cy3-labeled donkey anti-mouse, respectively. (A), (B) and (C) show the fluorescence signal captured, respectively, on the green, red and blue channels of the confocal microscope. (D-F) After a stripping procedure performed according to the protocol described in Material and Methods, the same section was re-stained with sheep anti-TH antibody and the same mouse anti-huαsyn antibody as before, then detected with the secondary antibodies Cy5-labeled donkey anti-sheep and Cy3-labeled donkey anti-mouse, respectively. The same cell as the one depicted in (A-C) was imaged in the three channels of the confocal microscope (D-F). (D) is the signal detected in the green channel and shows the absence of remaining fluorescence from the previous staining. (E) is the signal in the red channel and shows that after this stripping/re-staining procedure, the transferred huαsyn punctum (arrowhead) can still be detected. (F) is the signal in the blue channel and demonstrates that after stripping, the TH can be detected with an antibody different from the one used in the first staining. Scale bars, 5 µm. (TIF) [file pone.0039465.s003.tif]

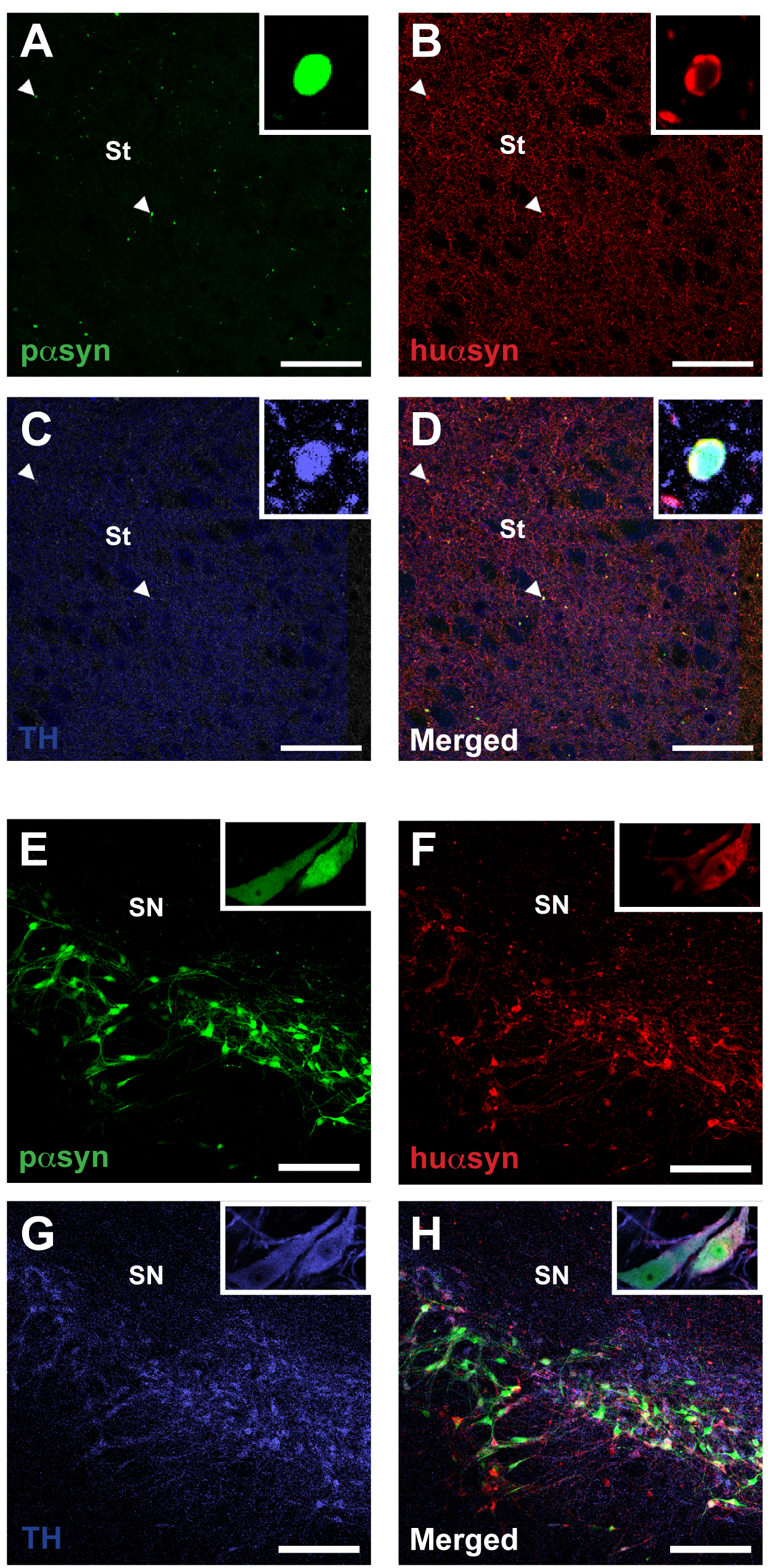

Supplement: Figure S4 — AAV2/6-huαsyn injected rats express phosphorylated α-synuclein both in the subtantia nigra and in the striatum. (A-H) Coronal sections from the striatum (A-D) or the substantia nigra (E-H) of a rat from the 3 week/4 week group, stained with antibodies directed against α-syn phosphorylated on serine 129 (green, pαsyn, A, E), human α-syn (red, B, F) and TH (blue, C, G). (D) and (H) are the merged pictures of (A), (B), (C) and (E), (F), (G), respectively. The arrowheads in (A-D) mark varicosities co-expressing pαsyn, huαsyn and TH. The insets in (A-D) and (E-H) show high magnification pictures of, respectively, a striatal varicosity and nigral dopaminergic neurons co-expressing the three markers. Abbreviations: St, striatum; SN, subtantia nigra. The scale bars equal 200 µm. (TIF) [file pone.0039465.s004.tif]
